# The Attitude of Great Writers Towards Disease

**Published:** 1892-10-01

**Authors:** 


### The Attitude of Great Writers Towards Disease.

*(Continued from page 357, Vol. XII.)*

XII.—DUMAS ON THE ART OF THE POISONER.

It is always amusing to trace the little predilections of novelists in the region of crime. They may vary their plots and catastrophes as they will, but there is nearly always to be noted a special element of mystery or violence which they have made their own, and in which they rejoice and are strong. Dickens was admirable in his big murders; Wilkie Collins breathed best in an atmosphere of sinister mystery; Trollope delighted in the poorer crimes of the respectable; Dumas was, beyond question, pre-eminent in the science and art of the poisoner.

It may, however, be disputed whether he had gained so complete a mastery over the subject in its scientific as in its artistic aspects. His acquaintance with the shady sides of mediæval Italian history was extraordinarily minute, and lent him a perfect grasp over the delicate processes attendant on a finely-conceived administration of the drugs. But it may be doubted whether the young clergyman who applied to Prince Florizel for books about "life" would have gained much practical information from Dumas about the art of committing crime. I incline to think the Prince was right in giving the preference to Gaboriau. For Dumas is either too vague or too grandiose for the ordinary criminal, and is apt to assume a command of resources out of the reach of all but Cardinals, Kings, and millionaires.

He tells us, for instance, that the poison of the Borgias was of two kinds: an impalpable powder, the composition of which is unknown, and a liquid prepared as follows—a strong dose of arsenic was administered to a wild boar, and at the moment when the poison was beginning to act, the animal was hung up by his heels. The foam which fell from his jaws was then collected in a silver plate, and transferred to a flask hermetically sealed. From beginning to end the process appears to offer grave difficulties to the vulgar.

Much of Dumas's poisoning must be referred back to the Borgias. It will be remembered that the fabulous riches of the Count of Monte Cristo dated back to the days when the notorious Alexander VI. held the Papal chair. Having determined to despoil the Cardinal Spada and his nephew of their vast possessions, the Pope followed his usual course on such occasions by inviting them to dinner. His son, the still more infamous Cæsar Borgia, "thought it might be well to use one of those means which he kept always at the disposition of his intimate friends. These were, first, the famous key with which certain persons were asked to open a certain cupboard. This key was furnished with a tiny steel point—negligence on the part of the workman. The guest had to press hard to open the cupboard, of which the lock was stiff, and thus received a prick from the little point of which he died the following day. There was also the lion-headed ring which Cæsar placed on his finger when he gave certain shakes of the hand. The lion 'bit' the skin of these favoured hands, and the bite was mortal at the end of twenty-four hours." The Pope, however, preferred on this occasion a simpler course, and the Cardinal and his nephew found their fate at his table in a dish of poisonous mushrooms. Expecting some such reception, however, the Cardinal had previously concealed his treasure in Monte Cristo's island, where it remained till Dantès took possession of it some centuries later. In his "Crimes Célèbres" Dumas recalls the singular directness of the fate which, according to the best tradition, overtook both Borgias in consuming themselves one thirsty day a beverage which had been prepared under their direction for one of their enemies.

Dumas had a keen eye in history for these striking revenges of fate. The only point of unity in the Reine Margot series lies in the career of François d'Alençon, culminating in the fine poisoning scene at the close of "Les Quarante-cinq." Here, again, he is stronger in the administration than in the preparation of the poison. We are indeed admitted to the laboratory in which Rémy has spent weeks in fabricating the poison. We see it distilled drop by drop and hermetically sealed in the solid glass flagon which alone can resist its corrosive action. But its composition is a mystery no less, it may be suspected, to the author than to the reader. The scene with which Diane works her long-accumulated revenge with it on the treacherous François is unsurpassed in Dumas' peculiar line of deliberate detail working up to a great dramatic effect. When the peach fails in its effect, when the poisoned rose stupifies only for a moment, the reader is roused to indignation against the cowardly assassin who is proof against his own favourite weapons. Then the torch is kindled, and as the smoke rushes back against the Prince, and he breathes the infected air never to breathe consciously again, the soul of the reader is satisfied as is Diane de Meridor with what she deems her lawful vengeance, and the wretched creature is abandoned without one touch of remorse, without one lingering shadow of blame on the executioner. It is not the highest morality, this 'tour de force' which reverses the judgment appropriated by the common consent of mankind to the poisoner and his victim. But fully to understand the propriety of this murder of Francois it is necessary to have followed the turbid course of his crimes in the volumes preceding the one in which they are ended. How well Dumas had studied that little group of the Medici blood who defiled the throne of France until Henry of Navarre could raise her lilies from the dust, and nowhere are the little family idiosyncracies of mutual hatred and treacherous intrigue more vividly depicted than in the series of plots and counterplots which led to the death of the unhappy Charles IX. We see Catherine de Medici lending her horrible ingenuity to smooth the path of her favourite and basest son François to the throne he was never to mount. Mother and son had resolved on the death of Henry of Navarre by a device no caution could frustrate. Well knowing Henry's passion for the chase (a taste he shared in common with Charles IX.) an ancient treatise on hunting, containing choice engravings, was prepared under Catherine's direction by the skill of her arch poisoner, René. Every leaf was heavily impregnated with arsenic and lightly gummed, so that it was necessary to wet the finger in order to turn the pages. The book was then carried by François himself to the apartment of Henry of Navarre, and left on the table to tempt his notice on returning from the hunt. But by a series of misadventures it was Charles who entered the room and bore off the volume, and all falling out as had been planned, it was the miserable King who, raising his finger to his mouth as each page resisted his touch, absorbed into his system the poison which his own mother had prepared and his own brother had administered. It is a terrible page of history which here lives again. But Dumas must be exonerated from the charge of immorality when he sets himself to show the fitting end of men like François.

It is a charge he does not always escape. Readers of "Monte Cristo" will remember the famous poisoning chapter in which the Count sets himself deliberately to instruct Madame de Villefort in the science of poisoning, to destroy her waning scruples, and finally to furnish her with the means of carrying out her designs. Here we have the interesting anecdote of the cabbage watered with arsenic, which was eaten by a fowl, which was eaten by a rabbit, which was eaten by a vulture, which fell into a stew-pond, where it was eaten by an eel, which was finally eaten by the very man it had been proposed to poison by this elaborate process. And here we have the mention of the celebrated Aqua Tofana, the secret of which is happily lost. But the intolerable airs of virtue and righteous judgment assumed by the Count cannot vindicate him from being the real author of the astounding series of murders by which Madame de Villefort, thus instructed, afterwards laid waste her husband's household. Just so far as the reader is induced to a kind of tacit acceptance of the situation, so far has the author laid himself open to a charge of the gravest immorality of which a writer can be guilty, viz., a blurring of the boundary line which eternally separates wrong from right.

